# Supplementary material for: Complexome profiling of the Chlamydomonas psb28 mutant reveals TEF5 as an early PSII assembly factor
Source: Plant Cell. 2025 Mar 17;37(6):koaf055. doi: 10.1093/plcell/koaf055 (PMC12164590; doi:10.1093/plcell/koaf055)
Supplement: koaf055_Supplementary_Data [file koaf055_supplementary_data.zip › psb28_interactive.zip!/plots/intensity/qualityPlots/peptides/CGLD9_Cre13.g569250.t1.2.html]

### Cre13.g569250.t1.2

Trivial name: CGLD9  
SeqCoverage [median]: 17.80  
Mass: 29.06 kDa  
  
 

| Sequence | MissedCleavage | Mass [Da] | wtIntensity | muIntensity | Score |
| --- | --- | --- | --- | --- | --- |
| GVPTAAATAAK | 0 | 956.53 | 0.00 | 1275.46 | 67.99 |
| PATAATPAGPR | 0 | 1008.54 | 270.57 | 0.00 | 75.38 |
| PGSVPRPTPPGGR | 1 | 1273.69 | 0.00 | 612.39 | 33.69 |
| SALAATTKPDEPDSLK | 1 | 1642.84 | 14129.93 | 20176.39 | 130.06 |
|  |  |  | 14400.5 | 22064.2 |  |
